# Macular Microvasculature Is Associated With Total Cerebral Small Vessel Disease Burden in Recent Single Subcortical Infarction

**DOI:** 10.3389/fnagi.2021.787775

**Published:** 2022-01-20

**Authors:** William Robert Kwapong, Shuai Jiang, Yuying Yan, Jincheng Wan, Bo Wu

**Affiliations:** Department of Neurology, West China Hospital, Sichuan University, Chengdu, China

**Keywords:** swept-source optical coherence tomography angiography, recent single subcortical infarction, magnetic resonance imaging, cerebral small vessel disease burden, macular microvasculature

## Abstract

**Purpose:**

To assess the retinal microvasculature, choriocapillaris, and choroidal thickness in recent single subcortical infarction (RSSI) patients compared with healthy controls. We also assessed the correlation between the macular microvascular changes and choroidal changes with their clinical implications in RSSI patients.

**Methods:**

Forty-six RSSI patients and 39 healthy controls (HC) were enrolled in our study. Magnetic resonance imaging (MRI) was done for all RSSI patients, and a total cerebral small vessel disease (CSVD) score was assessed for all patients. Swept-source optical coherence tomography (SS-OCT) was used to image and assess the choroidal thickness and SS-OCT angiography (SS-OCTA) was used to image and assess the macular microvasculature and choriocapillaris in all participants. Clinical information was collected for all participants.

**Results:**

RSSI patients showed significantly sparser inner retinal microvasculature (*P* = 0.003) when compared with healthy controls. RSSI patients showed significantly thinner choroidal thickness (*P* < 0.001) when compared with HC. No significant difference (*P* = 0.247) was seen when the choriocapillaris was compared between the two groups. CSVD burden (*P* = 0.014) and NIHSS score (*P* = 0.010) showed significant correlation with the inner retinal microvasculature of RSSI patients. The inner retinal microvasculature (*P* = 0.016) and choroidal thickness (*P* = 0.018) showed a significant correlation with the MoCA scores in RSSI patients.

**Conclusions:**

Our report suggests that retinal and choroidal imaging may serve as useful indicators to expand our understanding of RSSI and its clinical validity.

## Introduction

Recent single subcortical infarction (RSSI) formerly known as lacunar stroke accounts for a quarter of all ischemic strokes (Wardlaw, 2005); RSSI is usually caused by cerebral small vessel disease (CSVD) or steno-occlusion of a parent artery in the brain (Bladin and Berkovic, 1984; Jiang et al., 2020, 2021). It has been suggested that RSSI patients have early neurological deteriorations which may lead to poor functional outcomes (Jiang et al., 2021). As such, timely and effective diagnostic approaches become critical, as treatment is more likely to be successful in the early phase of the disease.

Eye-related disorders such as reduced vision and motion perception have been reported in patients with lacunar stroke (Rowe et al., 2009; Hepworth et al., 2021). Importantly, these eye-related disorders have been documented not only in the early phase of the disease but also occur simultaneously with the lesion in the brain (Rowe et al., 2019). To be specific visual abnormalities in lacunar stroke have been suggested to be linked with degeneration of the cerebral visual cortex which may translate to the posterior segment of the eye (Pula and Yuen, 2017).

Retinal imaging studies have shown that patients with lacunar stroke have significant changes in the retinal microvasculature and structure. Retinal fundus photography reports have shown thinner retinal arterioles and enlarged retinal venules in lacunar stroke patients when compared with healthy controls and suggested that these retinal microvascular changes may be pointers of microvascular pathology in the brain (Doubal et al., 2009; Lindley et al., 2009; Yatsuya et al., 2010). Yet very few reported on the retinal vascular changes and their correlation with the clinical implications. Following the advent of the optical coherence tomography angiography (OCTA), *in vivo* studies have shown significant changes in the retina and choroid of patients with neurodegenerative diseases such as Parkinson's disease (Robbins et al., 2021) and Alzheimer's disease (Li et al., 2021); these reports have suggested that the retina and choroid could be a potential early and non-invasive microvascular indicator for these diseases. However, very little is known about the clinical use of structural and microvasculature of the retinal capillary network and choroid in RSSI; this may be due to the persistent encounter of explicitly detecting the cause of RSSI clinically. Deviations in reported results of macular microvascular changes with RSSI have been shown to arise from intrinsic OCTA variabilities such as length of examination time and resolution for effectively visualizing the retina. Furthermore, previous reports used the fundus camera which can image the superficial vessels of the retina with less attention on the whole capillary network in the retina, choriocapillaris, and choroidal structure. Importantly, little is known about the association between the changes that occur in the retina and choroid and the clinical implications in RSSI patients.

Swept-source optical coherence tomography (SS-OCT) and swept-source optical coherence tomography angiography (SS-OCTA), the latest milestone in retinal imaging, is equipped with a longer wavelength to enable *in vivo* visualization of deeper-lying structures, has a faster scan speed which enables faster acquisition of B-scans therein allowing widefield scans and more accurate three-dimensional images of the retina and choroid. Our current study utilized the SS-OCT/SS-OCTA to assess the retinal microvasculature, choriocapillaris, and choroidal thickness in RSSI patients compared with healthy controls. We also assessed the correlation between the macular microvascular changes and choroidal changes with their clinical implications in RSSI patients.

## Methods

### RSSI Patients

Between December 2020 and August 2021, 50 recent single subcortical infarction patients were prospectively recruited from the neurology department of West China Hospital. Forty age- and gender-matched healthy controls were recruited as well. Written informed consent was obtained from all the participants or primary caregivers. The study was approved by the ethics committee of West China Hospital and was conducted according to the tenets of the Declaration of Helsinki.

The inclusion criteria for recent single subcortical infarction (RSSI) patients were as follows: (1) age 18–80 years old; (2) RSSI (no lesion diameter limit) in the perforator territory of the middle cerebral artery (MCA) or basilar artery (BA) identified using diffusion-weighted imaging performed within 7 days of symptom onset; (3) no relevant MCA or BA disease confirmed by magnetic resonance angiography (MRA). The exclusion criteria included: (1) previous history of stroke or transient ischemic attacks on the symptomatic side; (2) coexistent significant (≥50%) stenosis of the responsible extracranial large artery detected by computed tomographic angiography; (3) non-atherosclerotic vasculopathies, such as vasculitis, dissection, or Moyamoya disease; (4) presence of potential cardioembolic disorders such as atrial fibrillation, infective endocarditis, valvular heart disease, or current myocardial infarction identified on 24 h electrocardiographic or Holter monitoring, and transthoracic echocardiography; and (5) presence of retinal diseases on images such as diabetic retinopathy, glaucoma, or age-related macular degeneration (AMD). Healthy controls (HCs) were individuals who attended our hospital for an annual medical checkup; these individuals were not impaired in any cognitive domain during their cognitive assessment. These HC participants had no history or evidence of cerebrovascular disease or ocular abnormalities, neurological diseases, with a normal fundus and visual acuity.

Demographic and vascular risk factors including age, sex, education, body mass index (measured by the body mass divided by the square of the body height), hypertension, diabetes, hyperlipidemia, heart disease, and current smoking were collected. National Institutes of Health Stroke Scale (NIHSS) was measured at the time of admission. Trained neurologists performed cognitive assessment Montreal Cognitive Assessment (MoCA) on all participants.

### Magnetic Resonance Imaging Protocols and Imaging Analysis

**Figure 1:**
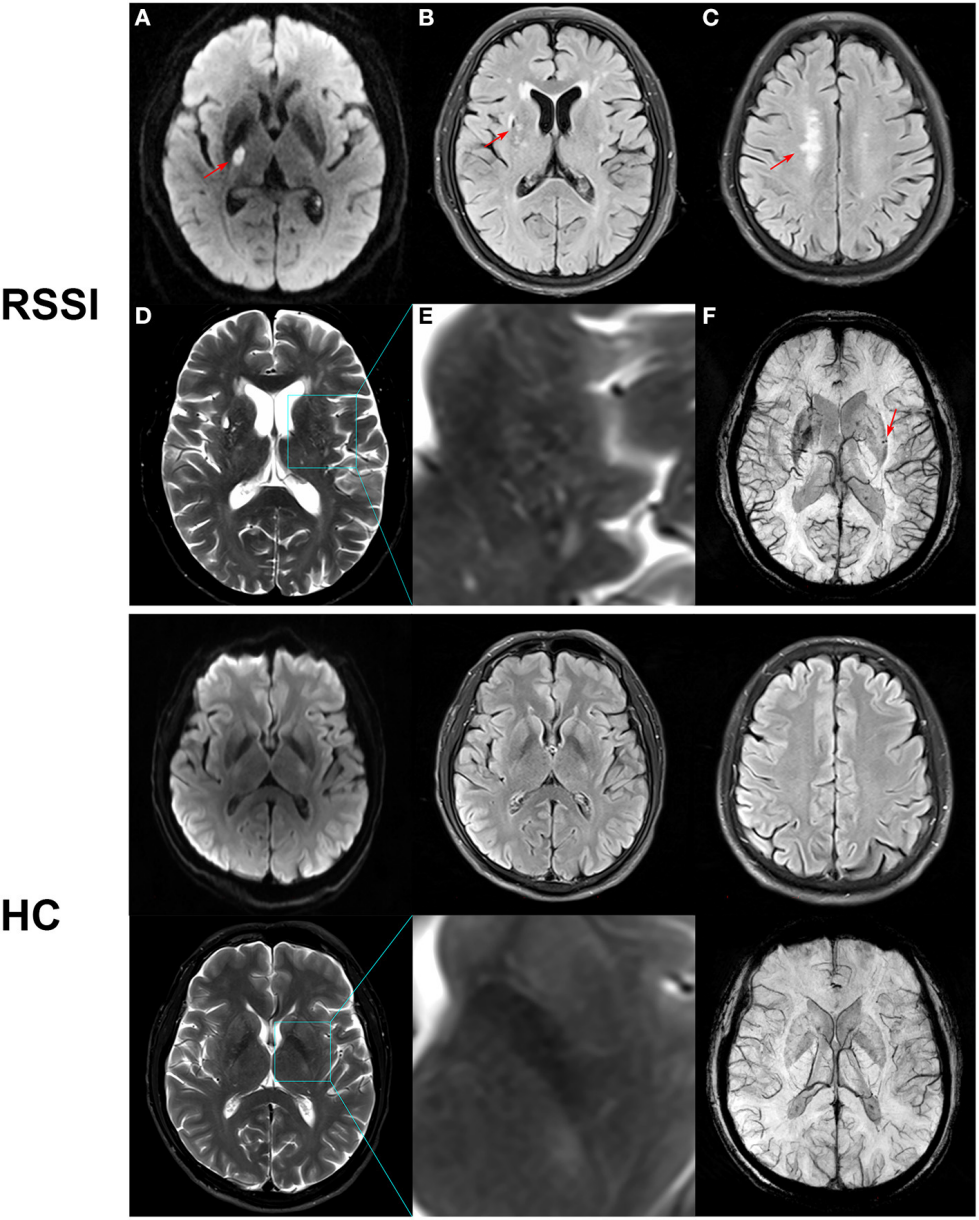
Representative MR images of recent subcortical infarction (RSSI) and healthy control (HC). Upper row: **(A)** DWI showed an acute single subcortical infarction in the right basal ganglia (arrow). **(B)** FLAIR showed a chronic lacune in the right basal ganglia (arrow). **(C)** FLAIR showed deep white matter lesions (arrow) with a Fazekas score of 2. **(D)** T2WI showed moderate enlarged perivascular space in the basal ganglia. **(E)** Magnified image of the outlined region indicated in **(D)**. **(F)** SWI showed cerebral microbleeds in the left basal ganglia. This patient had a total CSVD score of 4. Bottom row: A 55-year-old healthy control showed normal MR images with a total CSVD score of 0.

MR imaging was performed with a 3T Siemens Magnetom Trio system (Siemens, Erlangen, Germany) equipped with a 32-channel head coil. Diffusion-weighted imaging (DWI), three-dimensional time-of-flight MRA (3D-TOF-MRA), susceptibility-weighted image (SWI), and conventional Tl-weighted, T2-weighted, fluid-attenuated inversion recovery (FLAIR) images were obtained as shown in Figure 1. The detailed parameters of the imaging sequences have been well described in our previous study (Jiang et al., 2021).

All MR images were reviewed and analyzed with commercial software (OsiriX MD, Pixmeo SARL, Bernex, Switzerland) by two experienced readers (SJ and BW) blind to clinical information. If there was a discrepancy, a third decision was obtained.

Lacunes were defined as rounded or ovoid lesions involving the subcortical regions, 3–15 mm in diameter, of CSF signal intensity on T2 and FLAIR, generally with a hyperintense rim on FLAIR and no increased signal on DWI (Wardlaw et al., 2013). White matter hyperintensities (WMH) were defined as a high signal intensity region on the FLAIR sequence. The extent of periventricular and deep WMH was rated using the Fazekas scale (Fazekas et al., 1987) where periventricular WMH extends into the deep white matter (Fazekas score 3) or deep WMH (Fazekas score 2 or 3) was regarded as severe WMH.

Cerebral microbleeds (CMBs)were defined as homogeneous rounded hypointense lesions on susceptibility-weighted imaging with a diameter of 2-10 mm (Wardlaw et al., 2013). Enlarged perivascular spaces (EPVS) were defined as small (<3 mm) round or linear hyperintense lesions on T2 weighted images in the basal ganglia or centrum semiovale and rated as 0 to 4 on a validated semi-quantitative scale (Doubal et al., 2010). We only counted EPVS in the region of the basal ganglia which were specifically identified to be associated with cerebral small vessel disease (CSVD) (Doubal et al., 2010; Potter et al., 2015). An ordinal score ranging from 0 to 4 was constructed to reflect the total burden of CSVD, as previously described (Klarenbeek et al., 2013). The inter-observer agreement of measurements for each CSVD neuroimaging marker (lacunes, WMH, CMBs, and EPVS) was considered good to excellent.

### Swept-Source Optical Coherence Tomography and Swept-Source Coherence Tomography Angiography Imaging

The macula and choroidal images were obtained with SS-OCT/SS-OCTA (VG 200; SVision Imaging Limited, Luoyang China). The SS-OCT/OCTA system contained a swept-source laser with a central wavelength of 1050 nm and a scan rate of 200,000 A-scans per second. The imaging tool was equipped with eye-tracking software based on an integrated confocal scanning laser ophthalmoscope to eliminate eye-motion artifacts. 5 μm, 13 μm, and 3 mm were the axial resolution, lateral resolution, and scan depth respectively.

**Figure 2:**
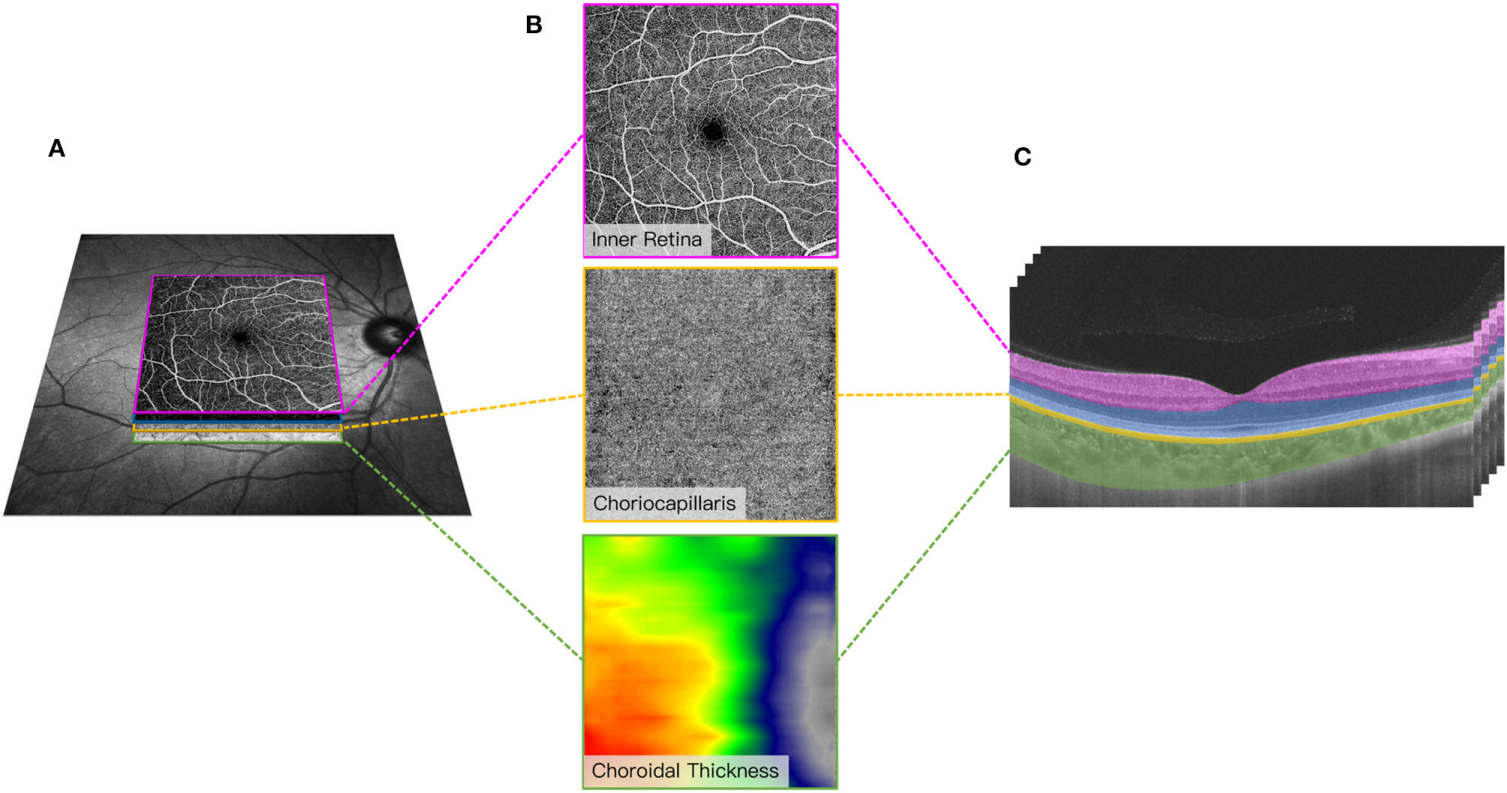
Representative 6 × 6 mm image of the macular microvasculature, choriocapillaris, and choroid centered on the fovea **(A)**. En face angiograms of the inner retina and choriocapillaris were generated by automatic segmentation in the OCT tool. A graphical representation of the choroidal thickness was shown to indicate the thickness. Warm colors indicate a thick choroidal structure while cold colors indicate a thin choroidal structure **(B)**. Segmentation of the inner retinal microvasculature, choriocapillaris, and choroidal thickness **(C)**. The inner retinal microvasculature was defined as microvessels found 5 μm above the inner limiting membrane (ILM) to 25 μm below the lower border of the inner nuclear layer (INL). The choriocapillaris was defined as the microvasculature from the basal border of the retinal pigment epithelium – Bruch's membrane complex to 20 μm below it. Choroidal thickness was measured from the base of the RPE – Bruch membrane to the choroidoscleral junction.

Fundus images of the OCTA were obtained with a raster scan protocol of 512 horizontal B-scans that covered an area of 6 × 6 mm^2^ centered on the fovea. The B-scans contained 512 A-scans. En face angiograms of the inner retina and choriocapillaris were generated by automatic segmentation to assess the retinal and choriocapillaris perfusions (Figure 2). The inner retinal microvasculature was defined as microvessels found 5 μm above the inner limiting membrane (ILM) to 25 μm below the lower border of the inner nuclear layer (INL). The choriocapillaris was defined as the microvasculature from the basal border of the retinal pigment epithelium – Bruch's membrane complex to 20 μm below it. Microvascular perfusion was used to assess the macula microvasculature and choriocapillaris; it was defined as the length of microvessels of the perfused choriocapillaris per unit area in millimeters squared (mm^2^) in the analyzed region (6 × 6 mm).

Choroidal thickness was measured from the base of the RPE – Bruch membrane to the choroidoscleral junction (measured in μm).

### Statistical Analysis

Participants' demographic variables were assessed using chi-square for categorical variables and unpaired *t*-test for continuous variables. SS-OCTA parameters were assessed using a generalized estimating equation (GEE) while adjusting for risk factors (age, gender, diabetes, hypertension, and dyslipidemia). Linear regression models were used to assess the association between SS-OCTA parameters and the clinical implication of RSSI patients. All data were expressed as the means ± standard deviations and were analyzed with SPSS (version 23; SPSS, Inc., Chicago, IL, USA).

## Results

Four RSSI patients and 1 HC were excluded from our data analysis because of the presence of age-related macular degeneration (*n* = 2) and severe glaucoma (*n* = 3). Forty-six RSSI patients (mean age = 56.50 ± 10.16 years) and 39 HCs (mean age = 55.62 ± 8.66 years) were included in our data analysis. Table 1 shows the demographics and clinical information of our enrolled participants. Out of the 46 RSSI patients, 12 patients (26.08%) had a CSVD score of 0, while 15 patients (32.61%) had 1, 9 patients (19.57%) had 2, 4 patients (8.70%) had 3 and 6 patients (13.04%) had 4. The mean NIHSS score of our enrolled RSSI patients was 3.70 ± 2.84. Importantly, RSSI patients (21.64 ± 5.92) had significantly reduced MoCA scores (*P* < 0.001, Table 1) compared with healthy controls (27.74 ± 1.08).

**Table 1:** Demographic and clinical information of study participants.

**Variable**	**SSI patients** **(***n =*** 46)**	**Healthy control** **(***n =*** 39)**	***P*** **value**
**Demographic information**			
Male sex, No. (%)	39 (84.78 %)	34 (87.18 %)	0.755
Age (years), mean (SD)	56.50 ± 10.16	55.62 ± 8.66	0.670
SBP, mmHg	144.23 ± 21.42	138.44 ± 14.64	0.172
DBP, mmHg	89.34 ± 13.55	84.67 ± 7.04	0.065
Hypertension, *n*	29	22	0.346
Diabetes mellitus, *n*	7	6	0.755
Duration, years	8.60 ± 2.61	6.17 ± 3.37	0.221
Dyslipidemia, *n*	15	11	0.974
Present smokers, *n*	18	20	0.962
Present drinkers (alcohol), *n*	18	15	0.529
NIHSS score	3.70 ± 2.84		
MoCA score	21.64 ± 5.92	27.74 ± 1.08	<0.001
Education, years	9.82 ± 4.50	9.29 ± 4.0	0.580
CSVD burden			
0	12 (26.08 %)		
1	15 (32.61 %)		
2	9 (19.57 %)		
3	4 (8.70 %)		
4	6 (13.04 %)		
Visual acuity, LogMAR	0.194 ± 0.176	0.001 ± 0.06	<0.001

### Comparison of SS-OCT/SS-OCTA Parameters Between RSSI and HC

RSSI patients showed significantly sparser inner retinal microvasculature (*P* = 0.003, Table 2) when compared with healthy controls. RSSI patients showed significantly thinner choroidal thickness (*P* < 0.001, Table 2) when compared with HC. No significant difference (*P* = 0.247, Table 2) was seen when the choriocapillaris was compared between the two groups.

**Table 2:** Comparison of optical coherence tomography angiography parameters between SSI patients and healthy controls.

**Variable**	**Mean (SD)**		**β Coefficient (95% CI)**	***P*** **value**
	**SSI patients** **(***n =*** 46)**	**Healthy control** **(***n =*** 39)**		
Inner retina (mm^2^)	2.32 ± 0.13	2.37 ± 0.10	−0.087 (−0.145–−0.029)	0.003
Choriocapillaris (mm^2^)	2.69 ± 0.16	2.76 ± 0.13	−0.031 (−0.084–0.022)	0.247
Choroidal thickness (μm)	321.31 ± 99.85	411.61 ± 84.33	−83.985 (−127.166–−40.804)	<0.0001

*Data were adjusted for blood pressure (systolic and diastolic), diabetes, dyslipidemia, age and gender*.

### Correlation Between Clinical Features and SS-OCT/SS-OCTA Parameters in RSSI Patients

CSVD burden (*P* = 0.014, Table 3) and NIHSS score (*P* = 0.010, Table 3) showed significant correlation with the inner retinal microvasculature of RSSI patients. The inner retinal microvasculature (*P* = 0.016, Table 3) and choroidal thickness (*P* = 0.018, Table 3) showed significant correlation with the MoCA scores in RSSI patients.

**Table 3:** Multivariate Regression Analysis between SS-OCTA parameters and clinical and radiological indicators of SSI.

**Variable**	**Inner retina**		**Choriocapillaris**		**Choroidal thickness**	
	**β Coefficient (95% CI)**	***P*** **value**	**β Coefficient (95% CI)**	***P*** **value**	**β Coefficient (95% CI)**	***P*** **value**
CSVD burden	0.067 (0.013–0.121)	0.014	−0.016 (−0.075–0.043)	0.594	15.503 (−18.453–49.459)	0.371
NIHSS score	−0.020 (−0.037–−0.003)	0.020	0.010 (−0.003–0.024)	0.120	1.985 (−3.732–7.702)	0.496
MoCA^†^	−0.013 (−0.024–−0.003)	0.016	0.009 (−0.010–0.029)	0.346	−5.655 (−10.36–−0.95)	0.018

*Data were adjusted for blood pressure (systolic and diastolic), diabetes, dyslipidemia, age, and gender*.

† *Data were adjusted for blood pressure (systolic and diastolic), diabetes, dyslipidemia, age, gender, and education*.

## Discussion

Our current study utilized the SS-OCTA to characterize the microvascular changes in the retina and choriocapillaris and used the SS-OCT to evaluate the structural changes in the choroid between RSSI patients and HCs. Our report showed that RSSI patients had significantly sparser inner retinal microvasculature and thinner choroidal thickness when compared with healthy controls. We also showed that these microvascular and structural changes significantly correlated with the clinical features of RSSI. Taken together, these findings suggest that the retina and choroid may be useful markers for assessing RSSI.

The pathogenesis underlying retinal microvascular changes in RSSI is vague. RSSI patients have been documented to have cerebral microvascular dysfunction compared with healthy controls (Knottnerus et al., 2009; Shi and Wardlaw, 2016). Previous retinal imaging reports using the fundus photographs showed that RSSI patients have significantly altered retinal vasculature when compared with healthy control and suggested that these retinal microvascular changes mirror the cerebral microvasculature changes that occur (Doubal et al., 2009; Lindley et al., 2009). However, fundus retinal imaging cannot visualize the deeper capillary network of the retina, and very little is known about these deeper microvascular changes. We showed that RSSI patients had significantly sparser inner retinal microvasculature when compared with healthy controls. The inner retinal microvasculature spans from the retinal nerve fiber layer (RNFL) to the outer plexiform layer and outer nuclear layer (ONL) and consists of the superficial vascular complex (SVC) and deep vascular complex (DVC) (Campbell et al., 2017). Therein our report suggests that RSSI affects the microvascular plexus of the inner retina. Contrarily, the incidence of atherosclerosis, a key factor in the pathogenesis of RSSI (Yaghi et al., 2021), in the retina of lacunar stroke patients (Yatsuya et al., 2010) suggests that neurodegeneration occurs in the retina concomitantly with cerebral structures. Moreover, it is suggested that the retinal microvascular is responsible for the metabolic supply of the neurons (Patton et al., 2005). As such, one may speculate that the retinal microvasculature in RSSI patients may reflect the underlying neurodegeneration with associated microvascular impairment.

Previous reports using the spectral-domain optical coherence tomography (SD-OCT) showed significant choroidal thinning in ischemic stroke patients when compared with the control group (Fang et al., 2017; Zhao et al., 2021). Our current study utilized the SS-OCT and showed RSSI patients had significantly thinner choroidal structure compared with healthy controls. Although the SD-OCT is useful in assessing the choroidal thickness, it uses a broadband near-infrared superluminescent diode as a light source which does not allow the permeation of light in the deeper layers of the choroid which obscures the boundary of the choroid thereby reducing the reliability of the choroidal boundaries (Miller et al., 2017). The SS-OCT has a faster scanning speed which allows for denser scan patterns and larger scan areas; also, it has a longer wavelength and its reduced sensitivity roll-off results in enhanced light penetration through the RPE as well as better detection of signals from deeper layers to overcome the barrier of the RPE (Miller et al., 2017). Our current study expanded previous studies by clearly defining the choroidal thickness as the distance between Bruch's membrane and the choroid-sclera interface (ie suprachoroid) (Wu et al., 2021). These boundaries measure the choroid extending as far as the vessel walls closest to the sclera. Thinning of the choroid in RSSI patients may involve mechanisms of thinning like those which occur in the choroidal plexus of the brain (Liebeskind and Hurst, 2004). The choroid plexus is a complex of capillaries that serves as a barrier against harmful substances and secretes various growth factors which help in the development of the brain (Javed et al., 2021). Likewise, the choroid in the eye is a vascular structure that serves as a barrier against harmful toxins and helps secrete various growth factors which help in the development of the retina (Nickla and Wallman, 2010). Ischemic stroke in animal models showed ischemic necrosis and damaged cells in the choroid plexus suggesting choroid plexus thinning during ischemic stroke (Xiang et al., 2017) which is congruent with our current finding. On the other hand, a previous on ischemic stroke and CADASIL suggested that choroidal thinning may be associated with atherosclerosis (Zhao et al., 2021).

Choroidal microcirculation accounts for more than 80% of ocular blood flow. The choroid consists of the choriocapillaris (capillary plexus of the choroid), Sattler's layer (consists of medium vessels), and Haller's layer (consists of large vessels) (Nickla and Wallman, 2010). However, whether RSSI has an impact on choroidal blood flow remains unknown. Using the SS-OCTA, our current study assessed the choriocapillaris perfusion and did not find any significant difference between RSSI and HC. Because of the large choroidal blood flow, an insult to the choriocapillaris may be insignificant due to the small area (about 10 μm) it occupies in the choroid (Nickla and Wallman, 2010). On the other hand, our RSSI patients were imaged within 7 days of symptom onset which may be a shorter period to have significant changes in the choriocapillaris. To the best of our knowledge, this is the first study to assess the choriocapillaris in RSSI, therein further reports may be needed.

The total cerebral small vessel disease (CSVD) score assesses the neuroimaging markers of CSVD (white matter lesions, presence of lacunes, periventricular spaces, and cerebral microbleeds); it mirrors the overall effect of CSVD on the brain. Clinically, the total CSVD burden differentiates the severity of CSVD (Cuadrado-Godia et al., 2018). Importantly, our report showed that the inner retinal microvasculature significantly correlated with the total CSVD score in RSSI patients. Accumulating reports have shown that the retina is susceptible to ischemic changes in the brain (London et al., 2013; Erskine and Herrera, 2014). Retinal imaging reports have shown that retinal microvascular impairment is associated with cerebral microbleeds (Qiu et al., 2008), lacunar infarction (Zhang et al., 2019) and white matter lesions (Cheung et al., 2010). Another recent report using the fundus camera showed an inverse correlation between cerebral white matter volume and arteriolar fractal dimension of the retinal vessels (McGrory et al., 2019). The studies used retinal vascular signs such as retinal microaneurysms, arteriovenous nicking, and retinal arteriolar/venular structure as their retinal vascular markers. However, these markers have been suggested to be late indicators of the disease mechanism (Patton et al., 2005). Our current study analyzed the retinal capillary changes which are sensitive to the earliest and small changes during the earliest pathological change that occurs. Based on the result of our study, quantified retinal microvasculature may serve as a structural indicator of CSVD burden with probable clinical use.

The National Institute of Health Stroke Scale (NIHSS) is widely used to evaluate the severity of an ischemic stroke and has been suggested to be a reliable predictive tool for stroke outcomes. Our previous report using the OCTA showed that SVC density correlated with NIHSS in participants with cerebral infarction and suggested that participants with higher NIHSS scores had severely damaged microvasculature in the SVC and vice versa (Kwapong et al., 2021). Likewise, the significant correlation between the inner retinal microvasculature which reflects the neuronal integrity of the retina and NIHSS suggests that microvascular impairment in the inner retina may reflect the neurological deficit in RSSI patients.

Noteworthy, we demonstrated the correlation between choroidal thickness and inner microvasculature and MoCA scores in RSSI patients. Visual stimulation is the main input for most neuropsychological examinations and the retina plays a significant role in visual processing in the central nervous system while the choroid helps in protecting and supplying blood to the photoreceptors (image forming cells). Simple computations for image analysis and explanations used in MoCA are done in the retina where previous studies have shown neurodegeneration and microvascular impairment. Although visual-related impediments in RSSI may be in part related to injury of the subcortical areas of the brain, the correlation between the retinal microvasculature/choroidal thickness in cognitive impairment in neurodegenerative diseases such as Alzheimer's disease has been reported while little is known in RSSI (Czako et al., 2020; Li et al., 2021). Our current report showing the correlation between retinal microvasculature and choroidal thickness and MoCA scores suggests that the inner retinal microvasculature and choroidal thickness may be useful to examine RSSI patients for potential cognitive impairment. Future studies and much stronger data showing that microvascular impairment predicts RSSI or CSVD and therefore MoCA would be needed to assess cognitive impairment. Furthermore, microvascular impairment and neurodegeneration in both the retina (Zhou et al., 2021) and choroid (Ikuno et al., 2010) may lead to visual loss (as shown in our current report) which has an impact on mental stimulation and social interactions – factors that have been reported to reduce the progression of cognitive impairment (Ruthirakuhan et al., 2012).

We acknowledge our study has several limitations. As with most imaging tools, participant cooperation is necessary. Patient movement can diminish the quality of images and some participants were excluded from the study because of movement during MRI and SS-OCT imaging. Also, choroidal thickness varies with age and axial length. Another limitation in our current study is we did not examine the axial length of our participants.

In conclusion, we demonstrated that RSSI patients have sparser inner retinal microvasculature and thinner choroidal structure compared with healthy controls. We also showed that sparser inner retina microvasculature correlated with NIHSS score, MoCA score, and CSVD burden score while thinner choroidal structure correlated with MoCA score in RSSI patients. Our report suggests that retinal and choroidal imaging may serve as useful indicators to expand our understanding of RSSI and its clinical validity.

## Data Availability Statement

The raw data supporting the conclusions of this article will be made available by the authors, without undue reservation.

## Ethics Statement

The studies involving human participants were reviewed and approved by Ethics Committee of West China Hospital. The patients/participants provided their written informed consent to participate in this study.

## Author Contributions

All authors listed have made a substantial, direct, and intellectual contribution to the work and approved it for publication.

## Funding

This work was supported by the National Natural Science Foundation of China (81870937, 82071320), and the 1.3.5 project for disciplines of excellence, West China Hospital, Sichuan University (No. ZYG D18009).

## Conflict of Interest

The authors declare that the research was conducted in the absence of any commercial or financial relationships that could be construed as a potential conflict of interest.

## Publisher's Note

All claims expressed in this article are solely those of the authors and do not necessarily represent those of their affiliated organizations, or those of the publisher, the editors and the reviewers. Any product that may be evaluated in this article, or claim that may be made by its manufacturer, is not guaranteed or endorsed by the publisher.
